# Social-Emotional Skills, Career Adaptability, and Agentic School Engagement of First-Year High School Students

**DOI:** 10.3390/ijerph20085597

**Published:** 2023-04-20

**Authors:** Íris M. Oliveira, Inês de Castro, Ana Daniela Silva, Maria do Céu Taveira

**Affiliations:** 1 Faculty of Philosophy and Social Sciences, Centre for Philosophical and Humanistic Studies, Universidade Católica Portuguesa, 4710 Braga, Portugal; imoliveira@ucp.pt; 2 Association of Psychology, University of Minho, 4710 Braga, Portugal; id10683@uminho.pt (I.d.C.); danielasilva@psi.uminho.pt (A.D.S.); 3 School of Psychology, University of Minho, 4710 Braga, Portugal

**Keywords:** career adaptation, psychosocial adjustment, high school

## Abstract

The transition to the first year of high school constitutes a critical moment because it corresponds to the implementation of a career choice, which can impact students’ satisfaction and psychosocial adjustment. The career construction model of adaptation holds potential to explain how students adapt to high school, by suggesting linkages among adaptive readiness, resources, responses, and results. However, research applying the career construction model to school transitions, combining social-emotional, career, and academic variables is still needed. This study explores the roles that social-emotional skills (an indicator of adaptive readiness) and career adaptability (an indicator of adaptability resources) play in explaining first-year high school students’ agentic school engagement (an indicator of adapting responses). Measures of social-emotional skills, career adaptability, and school engagement were completed by 136 students (63.2% girls; M age = 15.68). Results from the hierarchical linear regression analysis suggest that social-emotional skills and career adaptability explain 32% of the variance and significantly contribute to explaining agentic school engagement. These findings seem illustrative of the potential of the career construction model of adaptation to deepen knowledge and understanding about the transition to high school and the implementation of career choices. Aligned with the literature, this study supports the calls for integrative psychological practices that acknowledge social-emotional, career, and academic variables when fostering students’ psychosocial adjustment.

## 1. Introduction

In the Portuguese context, the transition from 9th to 10th grade is often challenging for adolescents, as it requires choosing and committing to a specific academic course to pursue in high school [1]. During this transition, adolescents face a formal and socially expected moment to make an academic choice and to think about their future more intentionally. Although several choices have been previously made during their lives, making a career choice by the end of middle school can either be exciting and relatively easy for some adolescents, or difficult and accompanied by doubt, insecurity, and anxiety for others [2]. Additionally, the transition from middle to high school cannot be separated from adolescent career development features. Adolescence is an important period of the lifespan, in which individuals explore and start consolidating intrapersonal attributes (interests, perceived competencies, values); identify and use exploratory resources to learn more about educational and labor options; advance in the construction of a vocational identity, by exploring, committing, and reconsidering tentative options, taking environmental barriers/opportunities, personal features and future goals into account [3,4,5].

Although both career choices and adolescent career development constitute classic topics of interest in the career psychology field, greater systematic attention must be paid to the implementation of career choices [6]. Three main approaches can be discerned in the literature on individuals’ career choices and decision-making process. One approach derives from developmental career theories and states that career choices are framed within an individuals’ developmental path and broader life-story [7,8]. Aligned with this approach, career development includes multiple cycles of planning and implementing choices throughout one’s lifespan. Agency, adaptability, and authorship need to be considered when addressing either expected or non-expected career choices, besides recognizing the meaning assigned to those choices within the individual’s career path [6,8]. A second approach relies on the prescriptive and descriptive models of the career decision-making process. It focuses on how career choices are made and how can they be most effective, relying on the steps required to solve career problems [9]. A third approach considers individual differences in career decision-making, thus identifying decision profiles and difficulties that often seem to occur in such a process [10,11]. Overall, these approaches call attention to the need to consider not only rational, but also emotional and self-regulatory aspects of the career decision-making processes, as well as the interplay between personal and contextual variables [4,5,6]. They also afford the possibility to conceive of career choice as a process that generally encompasses: a phase of identifying the need to make a choice, anticipate, and plan for a transition; another phase of executing the plan and implementing a decision. The latter phase has been less covered in research, although it needs to be considered to better respond to intervention requests and promote psychosocial adjustment [6].

In this regard, the career construction model of adaptation [12] has conquered attention over the last decade and holds potential to keep addressing career choices and transitions. Although the model has mostly been applied to transitions occurring during the adult period of the lifespan (e.g., [13]), it can also be considered in earlier age periods. It might be particularly helpful in investigating the implementation of career choices in the 10th grade and thus help explain how students adapt to high school. This model suggests a sequence of inter-related dimensions, namely adaptive readiness, adaptability resources, adapting responses, and adaptation outcomes [14,15]. 

Adaptive readiness consists of one’s willingness to change and to face the challenges inherent to career development tasks, occupational transitions, and work events [15]. Personality, hope, and optimism are among the constructs commonly conceived of as indicators of adaptive readiness [16]. Social-emotional skills can also serve the operationalization of adaptive readiness. Research, interventions, and policies targeting social-emotional skills have rapidly expanded in contemporary society, given its recognized importance in volatile, uncertain, complex, and ambiguous educational and work environments [17]. As various terminologies and perspectives can be found in the literature, the Organization for Economic Cooperation and Development (OECD) has advanced in an international and interdisciplinary efforts to consensually reach a definition and measure of social-emotional skills. The OECD developed a framework suggesting that social-emotional skills can be hierarchically organized in five broad dimensions (i.e., collaboration, task performance, engaging with others, emotional regulation, open-mindedness), each divided in various subdomains (e.g., tolerance, sociability) [18]. However, social-emotional skills should not be considered traits, since they have the potential to change and be developed throughout one’s lifespan [18,19]. Adolescence can be conceived of as a critical period in which social-emotional skills are developed and set the bases for the adoption of healthy habits, the embracement of intimate relationships, engagement in the community and in career self-management [20]. By developing social-emotional skills, adolescents are likely to present a more proactive stance anticipating problems, settings goals, and engaging in multicultural contexts, thus being better prepared to face challenges and attain academic, career, and personal life success [21,22,23]. There is evidence suggesting that social-emotional skills are positively associated with adolescents’ academic achievement, career decision self-efficacy, career exploration, and career adaptability [24,25,26,27]. 

Adaptability resources consist of psychosocial strategies or self-regulation resources needed to cope with life changes [15]. These resources can be aggregated in the general career adaptability concept, which “denotes an individual’s resources for coping with current and anticipated tasks, transitions, traumas in their occupational roles that, to some degree large or small, alter their social integration” [28] (p. 662). Adaptability resources are usually conceived in terms of the 4 Cs—concern, control, curiosity, and confidence [14,16]. Concern about the future considers the disposition to plan and prepare for future career steps [28]. Control consists of taking responsibility for one’s own choices, actions, and future, thus employing self-discipline, effort, and perseverance to attain goals [15]. Curiosity includes searching/collecting information about career alternatives and imagining the self in various possible roles and contexts [28]. Confidence considers one’s conviction to overcome barriers and to pursue aspirations [15]. Individuals that are concerned about their future assume control over their actions and career paths, demonstrate curiosity about their possible selves and occupational options, as well as feel confident in pursuing their goals are more likely to adaptively respond to social challenges and demonstrate psychosocial adjustment [13,14,15,16]. The overall career adaptability concept and the specific adaptability resources were subject to research in various contexts, populations, and periods of the lifespan, among which there is adolescence [29]. There are studies suggesting that adolescents’ career adaptability is positively associated with school engagement, career optimism, and life satisfaction, in addition to playing a protective role in minimizing career decision-making difficulties and mental health problems [30,31,32,33]. 

Adaptive responses include thoughts and behaviors that enable the individual to cope with changes and integrate into changeable contexts [15]. Career decision-making self-efficacy, career exploration, and career planning are often conceived of as indicators of adapting responses [14,16]. Although less frequently explored in this area, students’ engagement in school can also be conceived as an indicator of adapting responses. Engagement in school is a multidimensional concept that refers to a driving force linking students to school, that precedes academic adjustment and achievement, as well as minimizing risk behaviors and school dropout [34,35,36]. Although the cognitive, behavioral, and emotional/affective dimensions of engagement in school have been widely investigated, the agentic dimension has only been more recently considered [37,38]. Agentic engagement in school has captured increased attention in the scientific and practical community, as it considers students’ active participation in classes and agentic contribution to the learning process [36,37]. Research suggests that students’ agentic engagement in school is positively related with other engagement dimensions, academic achievement, motivation, basic psychological needs of autonomy, competence, and relatedness, self- and environment career exploration, as well as serves as a protective factor against test anxiety [38,39,40,41].

Adaptive readiness, adaptability resources, and adaptive responses jointly sustain the attainment of adaptation outcomes or results [14,15,16]. Career decidedness, commitment with a career choice, and well-being are usually taken as indicators of adaptation results [14,16]. Hence, acquiring more knowledge on previous career adaptation dimensions can help understand their inter-relations and sustain both research and empirical-based practices concerned with the promotion of individuals’ psychosocial adjustment. However, research applying the career construction model to school transitions as well as combining social-emotional, career, and academic variables is still scarce [42]. 

This study intends to explore the roles that social-emotional skills (an indicator of adaptive readiness) and career adaptability (indicator of adaptability resources) play in explaining first-year high school students’ agentic school engagement (indicator of adapting responses). Based on the career construction model of adaptation [13,15] and on extant research (e.g., [22,23,24,27,41]), the following research hypothesis was considered:

**Hypothesis** **1 (H1).** *Social-emotional skills and career adaptability can statistically significantly explain agentic school engagement.*

## 2. Method

### 2.1. Participants

The participants included 136 youths, 86 (63.2%) girls and 50 (36.8%) boys, aged 15–19 years old (*M* = 15.68, *SD* = 0.81). All participants attended 10th grade, with 92 (67.6%) being enrolled in scientific-humanistic courses, and 44 (32.4%) in professional courses. There were 27 (19.9%) students with previous retentions in their school paths. As for the families’ backgrounds, most of the youths’ mothers (65.4%) and fathers (79.4%) were employed at the time of this study. Most mothers (74.2%) and fathers (75%) held middle or high school as their completed educational levels. Most participants had siblings (83.1%). 

### 2.2. Measures

#### 2.2.1. Questionnaire of the Study on Social and Emotional Skills (SSES) 

The SSES is a large-scale international survey that assesses social-emotional skills. In the present study, following recommendations from the funding agency responsible for the psychometric evaluation of the measure in Portugal, 72 items answered in a five-point Likert-type scale, ranging from 1 (strongly disagree) to 5 (strongly agree) were used. These items enable the assessment of the following social-emotional skills, or subdomains, with eight items each: tolerance (e.g., “I learn a lot from people with differing beliefs”), trust (e.g., “I believe that most people are honest”), empathy (e.g., “It is important that my friends are ok”), responsibility (e.g., “I keep promises”), optimism (“I believe good things will happen to me”), curiosity (“I love learning new things in school”), assertiveness (e.g., “I like being a leader in my class”), cooperation (e.g., “I get along well with others”), and sociability (e.g., “I like talking to a lot of different people”). In the OECD survey, these skills or subdomains showed good internal consistency reliability, with values of Cronbach’s alpha ranging from 0.70 to 0.80 [18]. In this study, Cronbach’s alpha values ranged from 0.72 to 0.85. 

#### 2.2.2. Career Adapt-Abilities Scale—Portugal Form (CAAS) 

The CAAS—Portugal Form [28,43] includes 28 items answered in a five-point Likert-type scale ranging from 1 (not strong) to 5 (strongest). The items are equally distributed in four subscales inherent to the adaptability resources: concern (e.g., “Thinking about what my future will be like”), control (e.g., Taking responsibility for my actions”), curiosity (e.g., “Exploring my surroundings”), and confidence (e.g., “Working up to my ability”). Each subscale includes six items aligned with the CAAS international version and one additional item specific to the Portuguese setting [44]. The measure affords the possibility of obtaining a score for each subscale, and a total score indicative of career adaptability. The latter was considered for the purpose of this study. In the study of the CAAS adaptation and validation in Portugal, a Cronbach’s alpha of 0.92 was obtained for the total scale [44]. In this study, a Cronbach’s alpha of 0.95 was obtained. 

#### 2.2.3. Agentic Engagement Dimension from the Student Engagement in School: A Four-Dimensional Scale (SES-4DS) 

The SES-4DS [36] is a measure of students’ engagement in school. The agentic engagement dimension of the SES-4DS was used in this study to assess students’ active role in their learning processes and constructive participation in school. It includes five items (e.g., “During classes, I ask questions to my teachers”), answered in a six-point Likert-type scale from 1 (totally disagree) to 6 (totally agree). In the SES-4DS construction and validation study, a Cronbach’s alpha of 0.85 was obtained for the agentic dimension of school engagement [36]. In this study, a Cronbach’s alpha of 0.82 was obtained.

### 2.3. Procedures

This study is a part of a larger research project funded by the Portuguese Calouste Gulbenkian Foundation (Academy for Knowledge 240941). The project was scientifically and ethically approved by the Portuguese Calouste Gulbenkian Foundation and by the scientific board of the higher education institution hosting this study. 

The purpose and procedures of the project were presented to the principals and psychologists from nine schools located in northern Portugal, who approved their participation. School principals and psychologists selected the 10th grade classroom groups to which the project was presented. Written consent forms were obtained from the students’ caregivers and from the students themselves, thus assuring their voluntary, informed, and consented participation. Data were collected by the school psychologists in the classroom setting through an online application presenting the self-report measures. The students were all offered the same instructions, and the school psychologists provided additional clarification regarding the self-reported measures when needed. The ethical and deontological procedures established by the Portuguese Order of Psychologists, as well as by the Declaration of Helsinki, and the APA Ethical Principles of Psychologists and Code of Conduct were followed throughout this project. Individuals’ dignity and human rights, informed consent, volunteer participation, and confidentiality were assured.

### 2.4. Data Analysis

Data were analyzed with the Statistical Package for the Social Sciences (IBM SPSS), version 28 for macOS. Descriptive statistics were used to characterize the variables in the sample. Correlation coefficients were computed to explore the relations among the variables. The Kolmogorov–Smirnov and the Shapiro–Wilk normality tests were conducted previously to pursuing correlation coefficients [45,46]. 

Hierarchical linear regression analysis was used to test the contribution of social-emotional skills and career adaptability to explaining the variance in agentic school engagement. Relying on the career construction model of adaptation [12,15], the first step of the regression model included the social-emotional variables (i.e., tolerance, trust, empathy, responsibility, optimism, curiosity, assertiveness, cooperation, sociability) and the second step included career adaptability, assuming agentic school engagement as an outcome variable. Statistical assumptions were previously verified: the Durbin–Watson statistics suggested that residuals were uncorrelated; the variance inflation factor (VIF) and the tolerance statistic indicated no evidence of multicollinearity; no outliers were identified through Cook’s distance measure and the range of standardized residuals [46].

## 3. Results

Descriptive statistics results globally suggested moderate scores for social-emotional skills. Among them, the highest mean scores were found in cooperation and curiosity, whereas the lowest mean scores were obtained in assertiveness and responsibility. Moderate–high levels of career adaptability and of students’ agentic engagement in school were found, as the average scores were higher than the measures’ mean scores (Table 1). The Kolmogorov–Smirnov and the Shapiro–Wilk tests suggested that the social-emotional skills (except assertiveness) and the career adaptability scale variables presented a non-normal distribution. Hence, parametric (Pearson) and non-parametric (Spearman) correlation coefficients were performed. As the results from both parametric and non-parametric tests sustained the same conclusion regarding the retention or rejection of the null hypotheses, parametric results were presented [45]. 

The correlational results indicated that most of the social-emotional skills were statistically significantly correlated among them (although responsibility, assertiveness, and sociability were not correlated with some other social-emotional skills). Among the statistically significant correlations between social-emotional skills, most of them were positive, except the correlation between responsibility and cooperation, which was negative. Career adaptability was positively and statistically significantly correlated with the social-emotional skills, except with responsibility. In addition, students’ agentic engagement in school was positively and statistically significantly associated with following social-emotional skills: optimism, curiosity, and assertiveness, as well as with career adaptability (Table 2). 

As for the hierarchical linear regression model (Table 3), the first step explained 27% (adjusted *R^2^* = 0.22) of the variance of students’ agentic engagement in school and was statistically significant, *F* (9, 126) = 5.19, *p* < 0.001. Optimism consisted of a statistically significant variable explaining students’ engagement in school, with higher optimism being associated with higher agentic engagement levels, *β* = 0.43, *t* = 4.56, *p* < 0.001. The second step of the regression model explained 32% (Adjusted *R*^2^ = 0.27) of the variance and was also statistically significant, *F* (10, 125) = 5.94, *p* < 0.001. Optimism remained a statistically significant variable explaining agentic engagement, *β* = 0.34, *t* = 3.58, *p* < 0.001. Career adaptability also constituted a statistically significant variable explaining agentic engagement in school, *β* = 0.33, *t* = 3.09, *p* = 0.002. Hence, higher optimism and higher career adaptability are associated with higher agentic engagement in school. 

## 4. Discussion

This study relied on the career construction model of adaptation [12,15] to examine the roles of social-emotional skills and career adaptability in explaining first-year high school students’ agentic engagement in school. The results from this study partially supporting H1, as both the social-emotional skill optimism and career adaptability were shown to contribute to the explanation of agentic engagement in school. 

Among social-emotional skills, optimism was the one demonstrating a significant role in agentic engagement in school. Although it was expected that other social-emotional skills could help explain variations in agentic engagement in school, this finding highlighting optimism is aligned with career construction-based research suggesting its importance when addressing career processes and transitions [16], particularly among adolescents [32,33]. Optimism can translate a positive orientation towards the future, which has growingly been acknowledged as a personal resource to cope with expected and unexpected transitions in changeable educational and labor settings, as well as to sustain psychosocial adjustment and well-being [32]. Students demonstrating higher levels of optimism might be more likely to employ effort and perseverance in school tasks, as well as actively ask questions and embrace responsibility for their learning. It would be important for future studies to keep exploring the relations between optimism and academic processes. For example, linkages between optimism, learning self-efficacy, self-regulation, and value assigned to school could be addressed in further research. 

Nonetheless, it is also worth noticing that, against expectations, the remaining social-emotional skills did not offer a significant contribution for students’ agentic engagement in school. This finding may be related to the measure used herein to assess the social-emotional skills. Although the measure derives from interdisciplinary and international efforts to reach a consensus regarding the definition and measurement of social-emotional skills [18,43], it might be too general and not specifically aligned with the student role and the career development challenges. Hence, it would be important for future studies to keep analyzing the relations among social-emotional skills, career, and academic processes, employing measures of social-emotional skills more attuned with the academic settings and the career challenges. It might also be the case that the role of specific social-emotional skills varies according to the main phases of the career decision-making process. Future studies could explore this possibility, identifying whether there are social-emotional skills of greater importance in the phase of anticipating and planning for a transition, and in the phase of executing and implementing a decision [2,6], such as the one covered in this study. 

Additionally, this study suggested that career adaptability plays a significant contribution on students’ agentic school engagement. This finding is consistent with previous evidence illustrative of the inter-relations among career and academic processes [27,30,41,42]. It seems that students who are concerned about their future, eager to learn more about themselves and occupational options, confident and with a sense of control over their actions might better adapt to a school transition and actively contribute to improving their learning conditions. However, choosing and committing to a specific high school course is a complex process for adolescents and differently experienced by each individual [1,2]. On the one hand, there are adolescents who might have been actively preparing for the school transition by exploring the options and making an informed career decision; this may sustain high commitment and satisfaction upon the implementation of the career choice. On the other hand, there are adolescents who may have felt insecure about the transition and who may have made an uninformed choice; this may sustain a weak commitment and increase the likelihood of maladjustment when implementing the career choice, thus leading to reconsideration. 

## 5. Limitations and Conclusions

Despite the contribution from this study, five main limitations should be considered. First, a limitation regarding sample representativeness and size should be stated. This study relied on a sample of first-year high school students from northern Portugal, which is not fully representative of 10th graders from several Portuguese geographical areas. It is also worth noticing that although the sample size was aligned with some recommendations in the statistics literature [46], a larger sample would have been useful to increase the power of analysis. Hence, caution in the generalization of the results is warranted. It would be important to replicate this study with a larger sample of 10th graders representative of the various geographical areas of the country. The attainment of a larger and more representative sample would additionally afford the possibility to pursue structural equation models. Second, specific contextual variables from the schools which students attended were not controlled in the analysis. It could be useful for future studies to identify variables from the schools (e.g., public versus private, located in urban versus rural areas) that could potentially impact the covered variables [12,35] and control for their effect. Third, this study used a cross-sectional design, which does not allow inferring causality. Future longitudinal studies on the topic are required to better examine the inter-relations among the variables over time. Fourth, although this study advanced evidence on the indicators of adaptative readiness, adaptability resources, and adaptive responses in the moment of implementing a career decision, an indicator of adaptation outcomes (e.g., well-being) was missing. Future research could cover indicators of all dimensions of the career construction model of adaptation [14,15] to empirically test the fit of such a structural model among adolescents. Fifth, this study relied on data collected through self-report measures, which can be subject to a social desirability bias. Future studies could consider reports from others (e.g., teachers, parents) and use additional data collection techniques (e.g., classroom observation) to obtain complementary data.

Nonetheless, two main conclusions can be retrieved from this study. First, it illustrates the potential of the career construction model of adaptation [12,15] to theoretically sustain research regarding the implementation of a career decision and covering school transitions. As the career construction model has mostly been considered in studies with young adults and adults [e.g.,13,16], future research might be inspired by the possibility of applying this model when studying younger samples and earlier career transitions.

Second, this study evidenced that social-emotional skills (namely optimism) and career adaptability jointly contribute to explaining first-year high school students’ agentic engagement in school. This supports the need to consider social-emotional skills, career, and academic processes in an integrative manner, instead of in the fragmented and separate manner that has prevailed in both research and practice [20,42]. 

Various possibilities to keep pursuing this line of research and to enrich practice can be highlighted. For example, regarding research, future studies could employ a longitudinal design to analyze changes and reciprocal influences among social-emotional skills, career adaptability, and agentic engagement in school over time, employing structural equation models. Further research could also test the potential role of other variables that may complementarily help explain students’ agentic engagement in school (e.g., teaching methods, school climate). Additionally, given the importance that vocational identity development and career exploration, commitment, and reconsideration mechanisms assume in adolescence [3,4,5,9], future studies could explore whether the relation between career adaptability and agentic engagement in school remains across groups of adolescents varying in their stages of vocational identity development and in their levels of commitment to the chosen high school course. Such a research endeavor would be useful to deepen the scientific knowledge regarding the implementation of career choices [6], as well as to support the psychological practices targeting first-year high school students. Research could also test the efficacy of integrative psychological interventions, as it seems that, by promoting social-emotional skills and career adaptability, one can simultaneously foster students’ agentic engagement in school. 

As for practice, career education programs might be particularly useful to promote not only career competencies (e.g., career exploration, planning), but also personal and social competencies (namely social-emotional skills) that are jointly required to face the challenges of the current society [13,14,20,22,42]. By fostering these competencies, practitioners might help to better prepare students to adapt to life transitions [17,20,21,23], besides sustaining their engagement in school, which is critical for academic success [34,37]. These integrative efforts are aligned with a holistic and comprehensive view of the individuals [42] and can ultimately contribute to support their mental health and psychosocial adjustment [13,17,22,31,33], particularly in a challenging period such as those of implementing a career choice and starting high school.

## Figures and Tables

**Table 1:** Descriptive statistics results.

Variable	Mean (Standard Deviation)	Skewness	Kurtosis	Kolmogorov–Smirnov	Shapiro–Wilk
Tolerance	3.84 (0.48)	−0.68	2.67	0.09 **	0.96 ***
Trust	3.44 (0.44)	−0.86	3.36	0.12 ***	0.94 ***
Empathy	3.69 (0.40)	−0.43	2.02	0.10 **	0.96 ***
Responsibility	3.29 (0.38)	0.23	−0.67	0.09 **	0.97 **
Optimism	3.49 (0.42)	−0.51	0.08	0.14 ***	0.96 ***
Curiosity	4.01 (0.52)	−0.43	0.19	0.08 *	0.97 **
Assertiveness	2.57 (0.84)	0.07	−0.41	0.06	0.98
Cooperation	4.25 (0.40)	−0.64	1.47	0.10 **	0.95 ***
Sociability	3.40 (0.28)	0.07	1.51	0.12 ***	0.97 **
Career adaptability	4.00 (0.53)	−0.78	1.91	0.07	0.97 **
Agentic engagement	3.80 (0.98)	−0.14	−0.31	0.07	0.98

* p < 0.05. ** *p* < 0.01. *** *p* < 0.001.

**Table 2:** Correlation results.

Variable	1	2	3	4	5	6	7	8	9	10	11
1 Tolerance	1										
2 Trust	0.23 **	1									
3 Empathy	0.47 ***	0.23 **	1								
4 Responsibility	−0.02	0.05	−0.05	1							
5 Optimism	0.23 **	0.32 ***	0.32 ***	−0.02	1						
6 Curiosity	0.60 ***	0.20 *	0.20 *	−0.16	0.21 *	1					
7 Assertiveness	0.04	0.08	0.08	−0.01	0.44 ***	0.21 *	1				
8 Cooperation	0.43 ***	0.44 ***	0.44 ***	−0.20 *	0.44 ***	0.44 ***	−0.08	1			
9 Sociability	0.03	0.27 ***	0.27 ***	0.17 *	0.02	0.02	0.02	0.36 ***	1		
10 Career adaptability	0.38 ***	0.36 ***	0.36 ***	−0.16	0.54 ***	0.56 ***	0.26 *	0.53 ***	0.22 *	1	
11 Agentic engagement	0.11	0.11	0.13	0.02	0.46 ***	0.31 ***	0.24 **	0.16	0.11	0.46 ***	1

* p < 0.05. ** *p* < 0.01. *** *p* < 0.001.

**Table 3:** Results from the hierarchical linear regression model.

Explaining Variables	*R*^2^ (Adjusted *R*^2^)	*F*	β	*T*
Step 1 (Social-emotional skills)	0.27 (0.22)	5.19 ***		
Tolerance			−0.09	−0.89
Trust			−0.02	−0.22
Empathy			0.04	0.43
Responsibility			0.03	0.41
Optimism			0.43	4.56 ***
Curiosity			0.23	2.14 *
Assertiveness			0.13	1.49
Cooperation			−0.12	−0.93
Sociability			0.01	0.14
Step 2	0.32 (0.27)	5.94 ***		
Tolerance			−0.09	−0.83
Trust			−0.06	−0.63
Empathy			−0.02	−0.16
Responsibility			0.06	0.79
Optimism			0.34	3.58 ***
Curiosity			0.12	1.12
Assertiveness			0.08	0.99
Cooperation			−0.15	−1.26
Sociability			0.01	0.01
Career adaptability			0.33	3.09 **

* *p* < 0.05. ** *p* < 0.01. *** *p* < 0.001.

## Data Availability

The dataset supporting the results presented in this paper is available upon request from the corresponding author. As the project framing this study is still ongoing, the data are not yet publicly available.
